# Vitamin C deficiency in critically ill COVID-19 patients admitted to intensive care unit

**DOI:** 10.3389/fmed.2023.1301001

**Published:** 2023-12-20

**Authors:** Luis Chiscano-Camón, Juan Carlos Ruiz-Rodriguez, Erika P. Plata-Menchaca, Laura Martin, Ivan Bajaña, Cristina Martin-Rodríguez, Clara Palmada, Roser Ferrer-Costa, Silvia Camos, Yolanda Villena-Ortiz, Vicent Ribas, Adolf Ruiz-Sanmartin, Marcos Pérez-Carrasco, Ricard Ferrer

**Affiliations:** ^1^Intensive Care Department, Vall d’Hebron University Hospital, Vall d’Hebron Barcelona Hospital Campus, Barcelona, Spain; ^2^Shock, Organ Dysfunction and Resuscitation Research Group, Vall d’Hebron Research Institute (VHIR), Vall d’Hebron University Hospital, Vall d’Hebron Barcelona Hospital Campus, Barcelona, Spain; ^3^Departament de Medicina, Universitat Autonoma de Barcelona, Bellatera, Spain; ^4^Clinical Biochemistry Service, Vall d’Hebron University Hospital, Vall d’Hebron Barcelona Hospital Campus, Barcelona, Spain; ^5^Clinical Biochemistry Laboratory, ICS-IAS Girona Clinical Laboratory, Doctor Josep Trueta University Hospital, Girona, Spain; ^6^Fundació Eurecat Centre Tecnològic de Catalunya, Catalonia, Spain; ^7^CIBER Respiratory Diseases (CIBERES), Instituto de Salud Carlos III, Madrid, Spain

**Keywords:** SARS-CoV-2 pneumonia, acute respiratory distress syndrome, vitamin C, ascorbic acid, COVID-19

## Abstract

**Objectives:**

To determine vitamin C plasma kinetics, through the measurement of vitamin C plasma concentrations, in critically ill Coronavirus infectious disease 2019 (COVID-19) patients, identifying eventually the onset of vitamin C deficiency.

**Design:**

Prospective, observational, single-center study.

**Setting:**

Intensive Care Unit (ICU), Vall d’Hebron University Hospital, Barcelona. Study period from November 12th, 2020, to February 24th, 2021.

**Patients:**

Patients who had a severe hypoxemic acute respiratory failure due to COVID-19 were included.

**Interventions:**

Plasma vitamin C concentrations were measured on days 1, 5, and 10 of ICU admission. There were no vitamin C enteral nor parenteral supplementation. The supportive treatment was performed following the standard of care or acute respiratory distress syndrome (ARDS) patients.

**Measurement:**

Plasma vitamin C concentrations were analyzed using an ultra-performance liquid chromatography (UPLC) system with a photodiode array detector (wavelength set to 245 nm). We categorized plasmatic levels of vitamin C as follows: undetectable: < 1,5 mg/L, deficiency: <2 mg/L. Low plasma concentrations: 2–5 mg/L; (normal plasma concentration: > 5 mg/L).

**Main results:**

Forty-three patients were included (65% men; mean age 62 ± 10 years). The median Sequential Organ Failure Assessment (SOFA) score was 3 (1–4), and the Acute Physiology and Chronic Health disease Classification System (APACHE II) score was 13 (10–22). Five patients had shock. Bacterial coinfection was documented in 7 patients (16%). Initially all patients required high-flow oxygen therapy, and 23 (53%) further needed invasive mechanical ventilation during 21 (± 10) days. The worst PaO_2_/F_I_O_2_ registered was 93 (± 29). ICU and hospital survival were 77 and 74%, respectively. Low or undetectable levels remained constant throughout the study period in the vast majority of patients.

**Conclusion:**

This observational study showed vitamin C plasma levels were undetectable on ICU admission in 86% of patients with acute respiratory failure due to COVID-19 pneumonia requiring respiratory support. This finding remained consistent throughout the study period.

## Introduction

### Background

Among the most relevant critical care publications of recent years, research efforts on the role of vitamin C in critical illness have been outstanding. Research priorities have been the study of plasma kinetics, biological effects, and the potential of vitamin C as a treatment adjuvant in some subsets of critically ill patients.

Vitamin C is involved in the pathophysiology of the ischemia–reperfusion syndrome, immunomodulation, and inflammation (1) as it has antioxidant (2), anti-inflammatory, immune-enhancing effects and antiviral properties (3, 4).

The vitamin demonstrates direct virucidal activity and has effector mechanisms in both the innate and adaptive immune systems (5). Importantly, and with specific reference to the critical phase of COVID-19, vitamin C contributes to the downregulation of cytokines, protecting the endothelium from oxidant injury and has an essential role in tissue repair (6, 7). Vitamin C lessens reactive oxidative species (ROS) and inflammation via attenuation of NF-κB activation (8). Vitamin C significantly increases superoxide dismutase, catalase and glutathione and decreases serum TNFα and IL-1β levels in a rat ARDS model (9), while severe acute respiratory syndrome coronavirus 2 (SARS-CoV-2) downregulates the expression of type-1 interferons (the host’s primary anti-viral defence mechanism) (10), Vitamin C may also mitigate the increased risk of severe COVID-19 associated with upregulated ACE2, a receptor for the virus, and can prevent ACE2 upregulation induced by certain factors (11). Although there are many potential targets for vitamin C in the process of infection, viral replication and pathology in COVID-19, it is noteworthy that a key protease in the virus, Mpro, whose function is to activate several viral non-structural proteins, has been proposed as a target; ascorbate might be a powerful inhibitor of the enzyme (12). The critical and often fatal phase of COVID-19, may result in neutrophil migration and accumulation in the lung interstitium and bronchoalveolar space and is considered a key determinant of progression of ARDS (13). Neutrophil extracellular trap formation (NETosis) is a cell death pathway different from apoptosis and necrosis that traps and inactivates pathogens (14) and actually vitamin C is a novel regulator of NETosis (15). Furthermore, vitamin C enhances lung epithelial barrier function in an animal model of sepsis by promoting epigenetic and transcriptional expression of protein-channels at the alveolar capillary membrane that regulate alveolar fluid clearance which include cystic fibrosis transmembrane conductance regulator, aquaporin-5, the Na^+^/K^+^-ATPase pump and epithelial sodium channel (16).

Carr et al. showed that around 40% of critically ill patients with septic shock had low plasma concentrations of vitamin C. Half of non-septic critically ill patients also have hypovitaminosis C (17). This vitamin plays a critical role in reducing inflammation and preventing the systemic inflammatory response (2). Several studies have described that critically ill patients exhibit deficient vitamin C levels in plasma compared to healthy controls (18). This finding is not related to decreased dietary consumption. Hampl et al. used the Third National Health and Nutrition Examination Survey data to evaluate dietary, supplementary, and serum vitamin C on healthy out-hospital population, finding that the mean intake and serum concentrations of vitamin C were average (19).

Plasma vitamin C concentrations frequently decline to near-to-scurvy levels in patients with any condition characterized by overwhelming systemic oxidative and inflammatory stress, such as sepsis, trauma, burns, or major surgery (20–22). Vitamin C values also decrease in patients with ARDS and sepsis (23), and it has been estimated that a significant number of SARS-CoV-2 pneumonia develop ARDS (24, 25). Our previous pilot study was the first to describe the presence of vitamin C deficiency in COVID-19 patients. We studied plasmatic levels on 17 ± 1.7 day from ICU admission, finding that more than 90% of patients with ARDS due to COVID-19 presented undetectable concentrations of vitamin C (26). As patients were studied not from the beginning of ICU course, this study did not provide information about when the consumption of vitamin C occurred. Options may be diverse: perhaps prior to ICU admission? Perhaps prior to the symptoms onset? Then, a limitation of this previous study relies on the impossibility of determining the onset of vitamin C consumption, because it was a cross-sectional cut of incidence and the patients were studied on different days of evolution from their admission to the ICU. Knowing when vitamin C deficiency occurs can lead to improve selection criteria for future studies assessing the effectiveness of vitamin C administration in severe hypoxemic COVID-19 and critical care patients.

Since December 2019, evolving SARS-CoV-2 infection and pneumonia cases have caused a global state of emergency (27). The lack or unavailability of targeted treatments has focused efforts on supportive therapies (28). It has been hypothesized that vitamin C could mitigate the inflammatory cascade in ARDS patients (6, 29–31). Identifying the right timing for vitamin C levels measurement is relevant to identifying patients who could benefit the most from the replacement of vitamin C.

### Hipothesis

Vitamin C deficiency appears very early in patients with COVID-19 ARDS patients.

### Objective

To study vitamin C plasma kinetics, through the measurement of vitamin C plasma concentrations, in critically ill COVID-19 patients and identifying eventually when the onset of vitamin C deficiency occurs in this group of patients.

## Methods

### Study design and setting

A prospective single-center observational study of patients with severe COVID-19 pneumonia admitted to the ICU of Vall d’Hebron University Hospital, Barcelona, Spain, was conducted from November 12th, 2020, to February 24th, 2021. Patients who had a severe hypoxemic acute respiratory failure due to COVID-19 were included. Blood samples were obtained at ICU admission, day 5, and day 10 after ICU admission. The patients were collected in the COVID-19 ICU division.

### Inclusion and exclusion criteria

We included patients with COVID-19 pneumonia who required high flow nasal cannula (HFNC) oxygen therapy or invasive mechanical ventilation (IMV) and were admitted to the ICU. The exclusion criteria were pregnancy, life support limitation, patients with chronic kidney disease (CKD), kidney stone disease, and the impossibility of allowing the conservation of the plasma sample in an adequate way to not interfere in the vitamin C denaturation and interpretation.

### Analyzed data and scores

The data collected included demographic variables, past medical history, number of days between symptom onset and ICU admission, days on IMV or HFNC, lenght of ICU stay, worst P_a_O_2_/F_i_O_2_ ratio, the incidence of ventilator-associated pneumonia (VAP), need for rescue therapy and hypoxemia [neuromuscular blockade, prone position, extracorporeal membrane oxygenation (ECMO)], use of corticosteroid therapy and.renal failure. The severity of the disease was evaluated with the APACHE II (32) and SOFA scores (33).

Both scores were calculated using the worst parameters measured during the first 24 h of admission. The risk of in-hospital mortality of patients has been evaluated using the 4C Mortality Score (34). The search for bacterial coinfection was performed on admission to the ICU by means of sputum culture or bronchoalveolar lavage in addition to urine antigen test for *S.pneumoniae* and *L.pneumophila*. There were not only bacterial culture but also viral and fungal cultures performed. Regarding the ICU course, at any time in which deterioration in oxygenation or appearance of a new radiological infiltrate was suspected by analytical parameters, a microbiological test was repeated for bacterial and fungal culture in a sample of tracheal aspirate or bronchoalveolar lavage. Variables related to the treatment of SARS-CoV-2 pneumonia, and organ support measures were also analyzed. ARDS was defined according to the Berlin definition criteria (35). Data on the incidence of acute kidney injury (AKI) or failure, and the need for continuous renal replacement therapy (CRRT), were collected according to the latest Kidney Disease: Improving Global Outcomes (*KDIGO*) Clinical Practice *Guideline* criteria (36). The presence of septic shock or sepsis was defined according to the Sepsis 3 criteria (37). P_a_O_2_/F_I_O_2_ ratio was calculated using the worst values on mechanical ventilation. Also, the administration of high doses of methylprednisolone (from 1 to 2 mg/kg/day), the number of days on mechanical ventilation, length of ICU stay, and ICU and in-hospital mortality were registered. The study fulfilled the “Strengthening the reporting of observational studies in epidemiology (STROBE)” checklist for observational studies (38).

### Vitamin C analysis and sample extraction

Vitamin C is a highly volatile compound that can be easily oxidized and hydrolyzed (39). Following the recommendations published by Pullar *et al* (40), we performed the pre-analytical phase scrupulously (41). The protocol collected the blood sample before the infusion of pharmacological treatment, throughout a central venous catheter. Venous blood was collected into a 4 mL Vacuette® tube (lithium heparin), protected from light, cooled rapidly after collection, and immediately delivered to the laboratory. Two aliquots of plasma were separated in a centrifuge at 2.643 g for 10 min and stored frozen at −20°C, protected from light until their analysis (the sample has been analyzed within 7 days of arrival at the laboratory, since the stability of the vitamin C molecule is 7 days at −15°C). An equal volume of cold metaphosphoric acid was added to the samples while kept on an ice bath and protected from light exposure to extract and stabilize vitamin C from plasma. The samples were centrifuged, and the supernatants were transferred to autosampler vials. Plasma vitamin C concentrations were analyzed using an ultra-performance liquid chromatography (UPLC) system with a photodiode array detector (wavelength set to 245 nm). A volume of 2 μL was injected into an Acquity UPLC HSS T3 column. The method was fully validated in linearity (1.5–30 mg/L), precision (coefficient of variation <5%), and accuracy (bias <3%) (42). We defined plasmatic levels of vitamin C according to Hampl et al. (7) undetectable levels: < 1,5 mg/L, deficiency: < 2 mg/L; low plasma concentration: 2–5 mg/L; normal plasma concentration: > 5 mg/L.

### Statistical analysis

According to variable distribution, descriptive data were expressed as mean (standard deviation) or median (interquartile range, IQR, 25–75%). Categorical variables are expressed as number and frequency. The evolution of ascorbic acid was assessed with a Fisher Exact test for the different time points. The outcome for ascorbic acid was assessed using the Wilcoxon-Mann–Whitney two-sample rank-sum test for all the comorbidities. We performed a study with a logistic regression model fitting the ascorbic acid and using the different comorbidities as predictors for the three different time-points.

The sample size was calculated with a MANOVA model with repeated measures within factors with the statistical package G-Power. We selected an effect size of 0.2, an alpha error probability of 0.05, a power of 0.9, 3 measurements, and a correlation between measurements of 0.7. We also planned a 20% drop-off, which yield a sample size of 42 patients. *A posteriori* analysis with a population of 43 patients and correlation between measurements of 0.67 yielded a power of 0.92 for our study.

### Ethics statement

We complied with the guidelines for human studies. The procedures were followed in accordance with the ethical standards of the responsible committee on human experimentation. The research was conducted ethically following the World Medical Association Declaration of Helsinki (1975). Information revealing the subject’s identity was avoided. The study was approved by the local Clinical Research Ethics Committee (Clinical Research Ethics Committee (CEIm) of Vall d’Hebron University Hospital) [PR(AG)687/2020] with exemption from informed consent. The committee accomplishes both in its composition and in the Standard Work Procedure (SWP) with the Best Clinical Practice (BCP) standards (CPMP/ICH/135/95) and with Royal Decree 1090/2015. The datasets used and analyzed during the current study are available from the corresponding author on reasonable request. The authors declare that they have no competing interests. There was no fund reception. All authors were involved in providing care for the patient and they were all involved in writing and reviewing the manuscript. There were no acknowledgments, there were no contributions from individuals or organizations.

## Results

### Characteristics of the study population

A total of 43 patients were included (65% men; age 62 ± 10 years). The most common comorbidity was diabetes mellitus. The time of presentation (symptom onset) was 8 (5–10) days before ICU admission and the time from hospitalization to ICU admission was 2 (0–4) days. There were no patients in our sample who received SARS-CoV-2 vaccination previous to the hospitalization. Regarding its characteristics, the 4C mortality score was 11 (7–12), which confers a risk of in-hospital mortality of 10 to 35%. Other baseline characteristics are detailed in Table 1.

**Table 1:** Clinical characteristics of the critically ill COVID-19 patients.

Variable	COVID-19 ICU patients (*n* = 43)
Age (years)	62 ± 10
Male gender (*n*, %)	28 (65)
SARS-CoV-2 vaccination	0 (0)
Comorbidities (*n*, %)	
History of smoking	8 (19)
COPD	1 (0.02)
CKD	6 (14)
Liver Cirrhosis	1 (0.02)
Malignancy or immunosuppression	7 (16)
Diabetis mellitus	12 (28)
Hypertension	5 (12)
Hospital admission in the past year	10 (23)
Time from onset of symptoms to ICU admission (median, IQR; days)	8 (5–10)
Time from hospitalization to ICU admission (median, IQR; days)	2 (0–4)
SOFA (median, IQR)	3 (2–5)
APACHE II (median, IQR)	13 (10–22)
4C Mortality Score for COVID-19 (median, IQR)	11 (7–12)
Septic Shock (*n*, %)	5 (12)
Sepsis (*n*, %)	10 (23)
Bacterial coinfection on ICU admission (*n*, %)	7 (16)
Respiratory support	
High flow nasal cannula (*n*, %)	43 (100)
Invasive mechanical ventilation (*n*, %)	23 (53)
VV-ECMO (*n*, %)	0 (0)
ARDS variables	
PaO_2_/F_I_O_2_ ratio (mmHg)	93 (29)
Neuromuscular blockade (during ICU admission) (*n*, %)	23/23 (100)
VAP	19/23 (83)
Prone position (during ICU admission) (*n*, %)	21/23 (91)
*Analytical parameters*	
Day 1	
Leukocyte count (x10e9/L, median, IQR)	10,4 (6,4-13,1)
Platelet count (x10e9/L, mean, ED)	258 (97)
D-dimer (μg /ml, median, IQR)	448 (308–2,548)
Fibrinogen (g/L, median, IQR)	5,2 (4,5 – 6,3)
CRP (mg/dl, median, IQR)	9 (4,8 – 16,1)
LDH (U/L, mean, ED)	424,4 (145) U/L
Ferritin (μg /L, median, IQR)	990(460,1–1,497,3)
IL-6 (pg/ml, median, IQR)	41,7 (8,3–80.1)
Day 5	
Leukocyte count (x10e9/L, median, IQR)	10.6(7.3–15.02)
Platelet count (x10e9/L, mean, ED)	273 (119)
D-dimer (μg /ml, median, IQR)	952 (395–2,649)
Fibrinogen (g/L, median, IQR)	4,9 (4,3 – 5,8)
CRP (mg/dl, median, IQR)	4,5 (1,1 – 8,5)
LDH (U/L, mean, ED)	346 (223)
Ferritin (μg /L, median, IQR)	767 (443–1,450)
IL-6 (pg/ml, median, IQR)	13,9 (8.5–44)
Day 10	
Leukocyte count (x10e9/L, median, IQR)	10,5 (7.23–16)
Platelet count (x10e9/L, mean, ED)	261 (130)
D-dimer (μg /ml, median, IQR)	560 (365–1,362)
Fibrinogen (g/L, median, IQR)	5.53 (4.2–6.8)
CRP (mg/dl, median, IQR)	7.6 (2.14–16.6)
LDH (U/L, mean, ED)	316 (191)
Ferritin (μg /L, median, IQR)	739 (479–1,068)
IL-6 (pg/ml, median, IQR)	26.4 (11.3–181.5)
*Dietary intake*	
Day 1	
Oral intake (*n*, %)	4/43 (9)
Enteral nutrition (30 mL/kg/d iBMI) (*n*, %)	1/43 (2)
Day 5	
Oral intake (*n*, %)	13/41 (31)
Enteral nutrition (30 mL/kg/d iBMI) (*n*, %)	3/41 (7)
Parenteral nutrition (*n*, %)	16/41 (39)
Day 10	
Oral intake (*n*, %)	13/34 (38)
Enteral nutrition (30 mL/kg/d iBMI) (*n*, %)	8/34 (23)
Parenteral nutrition (*n*, %)	12/34 (35)
Renal failure during ICU admission	
AKI (*n*, %)	10 (23)
CRRT (*n*, %)	5 (12)
Outcomes	
Duration of mechanical ventilation (days)	21 (10)
Length of ICU stay (days)	15 (11)
ICU outcome (survivors) (*n*, %)	33 (77)
Hospital outcome (survivors) (*n*, %)	32 (74)

The worst PaO2/FIO2 values during the first 24 h of ICU admission are presented. AKI, acute kidney injury; APACHE II, Acute Physiology and Chronic Health disease Classification System II; ARDS, acute respiratory distress syndrome; CKD, chronic kidney disease; COPD, chronic obstructive pulmonary disease; CRT, C-reactive protein; CRRT: continuous renal replacement therapy; iBMI, ideal body mass index; ICU, intensive care unit; IL, interleukin; LDH, lactate dehydrogenase; SOFA, sequential organ failure assessment; VAP, ventilator-associated pneumonia; VV-ECMO, venovenous extracorporeal membrane oxygenation.

**Figure 1:**
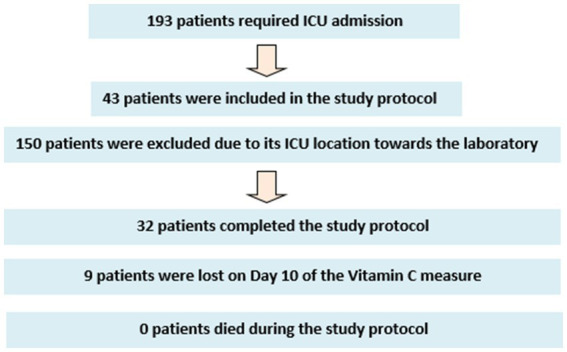
Patient flow-chart during the study protocol.

During the study period, a total of 197 patients required admission to the ICU. Of these, 43 have entered the study protocol. This offset between admitted and included patients derived from the location of patients. Due to the type of analysis that the vitamin C sample needs in order to not denature, the patients were recruited from the ICU hospitalization units close to the laboratory. Due to the sectorization of COVID-19 patients in our center, the vast majority of patients were referred to a sector where only patients with COVID-19 pneumonia were isolated and which was placed in a location that did not allow the conservation of the plasma sample in an adequate way. There were 9 patients in whom it has not been possible to measure the plasmatic concentration of Vitamin C on day 10 because they are patients who had already been discharged from the hospital (Figure 1).

### Organ dysfunction, respiratory support, and ICU complications

All patients received respiratory support with HFNC, and 23 (53%) subsequently needed IMV. All IMV patients fulfilled Berlin criteria for severe ARDS and received neuromuscular blockade. Twenty-one (91%) patients were placed in the prone position due to refractory hypoxemia. The primary organ dysfunction was respiratory, and the median SOFA score was 3 (2–5) points. Five (12%) patients presented septic shock, and 10 (23%) had sepsis. Seven (16%) patients had bacterial coinfection at ICU admission, and 83% developed VAP. There was no need for ECMO support.

### Vitamin C plasmatic levels

Low or undetectable levels remained constant throughout the study period in the vast majority of patients. Table 2 shows the evolution of plasma levels of vitamin C (days 1, 5, and 10 of ICU admission) and a breakdown of the plasma levels in the patients studied.

**Table 2:** Plasma concentrations of vitamin C at days 1, 5 and 10 of ICU admission.

	Plasma vitamin C concentrations (mg/L)		
Patient	VC - D1	VC - D5	VC - D10
**1**	<1.5	<1.5	<1.5
**2**	<1.5	<1.5	<1.5
**3**	<1.5	<1.5	<1.5
**4**	<1.5	2.3	NA
**5**	<1.5	<1.5	<1.5
**6**	<1.5	<1.5	<1.5
**7**	<1.5	<1.5	<1.5
**8**	<1.5	<1.5	<1.5
**9**	<1.5	<1.5	<1.5
**10**	<1.5	2.5	NA
**11**	<1.5	<1.5	<1.5
**12**	<1.5	<1.5	<1.5
**13**	<1.5	<1.5	<1.5
**14**	<1.5	<1.5	<1.5
**15**	<1.5	<1.5	<1.5
**16**	2.2	<1.5	<1.5
**17**	1.7	<1.5	<1.5
**18**	<1.5	<1.5	NA
**19**	<1.5	NA	2.6
**20**	1.8	NA	<1.5
**21**	<1.5	NA	<1.5
**22**	<1.5	<1.5	<1.5
**23**	<1.5	<1.5	<1.5
**24**	<1.5	<1.5	NA
**25**	<1.5	<1.5	<1.5
**26**	2	<1.5	<1.5
**27**	3.6	<1.5	<1.5
**28**	<1.5	<1.5	<1.5
**29**	4.5	NA	1.8
**30**	<1.5	2.2	NA
**31**	<1.5	2.2	NA
**32**	<1.5	<1.5	<1.5
**33**	<1.5	<1.5	<1.5
**34**	<1.5	NA	<1.5
**35**	<1.5	NA	<1.5
**36**	<1.5	<1.5	<1.5
**37**	<1.5	2.1	2.1
**38**	<1.5	NA	<1.5
**39**	<1.5	NA	<1.5
**40**	<1.5	<1.5	NA
**41**	<1.5	<1.5	<1.5
**42**	<1.5	<1.5	NA
**43**	<1.5	<1.5	NA

Reference range: undetectable levels: <1.5 mg/L; deficiency: <2 mg/L; low plasma concentration: 2–5 mg/L; normal plasma concentration:> 5 mg/L; VC: vitamin C; D1-5-10: day 1–5-10. NA: not available.

Upon ICU admission, all patients presented vitamin C deficiency: 37 of 43 patients (86%) even showed undetectable concentrations and 6 of them had low plasma levels. There were no patients who had levels in range at ICU admission.

There are some cases that deserves attention. At Day 5, where a large majority (83%) persisted with undetectable levels, there were five patients among which vitamin C levels increased to the low range (1.5 to 2 mg/L), despite being undetectable (<1.5 mg/L) on admission. These patients had single respiratory failure [mean SOFA and APACHE II scores of 2 (1) and 13 (6) points, respectively] and presented shorter ICU stays [6 (2) days]. None of them required invasive mechanical ventilation or presented shock during their ICU stay.

On day 10, 88% of patients had undetectable levels of vitamin C. Five out of nine patients who had been discharged from the ICU on day 10 had had low vitamin C levels on day 5, previously being categorized in an undetectable vitamin C range.

The evolution of vitamin C was assessed for the different time points with no significant differences detected. The value of p between 1 and 5 days and 1 and 10 days was 1 whilst the value of p between 5 and 10 days was 0.36. This result is in line with the evolution of ascorbic acid observed during the study.

The outcome for vitamin C was assessed using the Wilcoxon-Mann–Whitney two-sample rank-sum test for all the comorbidities. This test yielded a *p* > 0.05 for all subpopulations and time points. The logistic regression model showed for Day 1, apart from comorbidities at admission, all predictors were non-significant (p > 0.05). However, the 95% CI for this factor was [0.09, 4.41] and thus, we can conclude that it has no contribution to the prediction with a non-negligible probability. The behavior of this factor is observed for all comorbidities at 5 and 10 days. Thus, it can be concluded that the comorbidities observed do not present statistical significance in the time evolution of vitamin C.

### Outcomes

The mean duration of mechanical ventilation was 21 (10) days. The ICU length of stay was 15 (11) days. ICU and hospital survival were 77 and 74%, respectively.

## Discussion

The main contribution of the present study is that most critically ill COVID-19 patients admitted to ICU for severe acute hypoxemic respiratory failure present undetectable or low levels of vitamin C from the beginning of ICU course, and this finding remained unchangeable during ICU course. This data suggests that vitamin C plasmatic levels depletion occurs at some earlier time before ICU admission. We describe the best timing for collecting blood samples and the main clinical characteristics and severity of the patients studied. We believe that these findings are going to have important implications for patient selection in future interventional studies.

The relevance of vitamin C for the clinical course of patients with acute respiratory infection has been previously hypothesized. During infection, the generation of proinflammatory cytokines soon after the disease onset sets the enhancing environment for the development of multiorgan dysfunction (43). The “cytokine storm” leads to neutrophil migration and accumulation within the lung interstitium and bronchioalveolar space, a key event for progression in ARDS (13). There is evidence suggesting that vitamin C regulates this process (15). Possible mechanisms leading to vitamin C deficiency are increased metabolic consumption due to the enhanced inflammatory response, glomerular hyperfiltration, decreased gastrointestinal absorption, or reduced recycling of dehydroascorbate to vitamin C (44).

This present observational study showed vitamin C plasma levels were undetectable in the first 24 h after ICU admission in most critically ill COVID-19 patients admitted for acute respiratory failure requiring HFNC or IMV and remained invariable throughout the study period in most patients. Our group previously evidenced vitamin C deficiency in patients with severe COVID pneumonia (26). However the patients were studied on average on day 17.5 ± 1.7 from admission to the ICU, therefore, it was not possible to determine when the vitamin C deficiency occurred. That study was performed on 18 mechanically ventilated COVID-19 patients. Seventeen (94.4%) patients had undetectable levels of vitamin C, and one patient had low levels (2.4 mg/L). We could not determine the baseline levels at ICU admission as it was only a pilot study of the status of vitamin C in COVID-19 patients, being that one its main limitation. In that study, 94% of patients were placed in prone position due to refractory hypoxemia. In this new study, the patients were studied on admission to the ICU and the results were the same.

Reduced plasma vitamin C concentrations in patients with COVID-19 have been also described in previous small observational studies although with limited clinical information, as we quote as follows. In a previous report, Arvinte *et al* studied 21 critically ill patients. They found that 70% of patients had vitamin C deficiency. An important limitation of the study was that the authors did not register any clinical parameters, and the severity of respiratory failure was not associated with low plasma vitamin C levels (45). Xing et al. studied 31 hospitalized patients with COVID-19 and 51 healthy controls. The mean plasma vitamin C concentrations in the 8 patients with COVID-19 who were not supplemented with vitamin C were 2.00 mg/L (reference range: 0.5–4.90 mg/L), almost 5-fold lower than healthy volunteers. The severity of organ dysfunction was not assessed (46). Muhammad et al. compared 50 COVID-19 patients with 21 healthy controls in a population from Jigwa, Nigeria (47). The COVID-19 patients had statistically lower vitamin C plasma levels (3.3 mg /L) than healthy controls (4.4 mg /L). However, clinical parameters and organ dysfunction scores were not reported. Pincemail *et al* described 9 critically ill patients with severe COVID-19 pneumonia who had mean vitamin C plasma levels of 3.8 mg/L (48). The critically ill population of this study had significant comorbidities and a respiratory dysfunction requiring mechanical ventilation. To highlight, it has not been clarified when the sample for vitamin C analysis was obtained in these previous studies. In another report (49), vitamin C plasma levels were evaluated in 67 COVID-19 patients who accomplished ARDS criteria. Fifty-five (82%) patients had values <4 mg/L, and 12 patients (18%) had values of <1 mg/L. The blood samples were obtained during the first 24 h after ICU admission. Of note, the detection threshold was 1.5 ± 0. 5 mg/L. Consequently, 65 patients (82%) would have had undetectable levels according to the detection threshold of the our present study. However, this case series was not comparable with ours because the population the studied was milder than ours. Sinnbert et al. (50) recently studied 74 patients of whom 27 were cataloged as those with the most severe impairment.They found that COVID-19 patients had significantly lower plasma vitamin C levels than the controls. However, it is not specified at what stage of the evolution of the viral infection the serum sample was taken for ascorbic acid analysis and no variables of oxygenation or severity of hypoxemic respiratory failure were described. Hafez et al. (51) described a population where only 7 out of 67 patients were admitted to the ICU and where 58.2% of COVID-19 patients had deficient vitamin C levels. The risk of COVID-19 severity decreased in patients with vitamin C levels in range by 52% compared to patients with vitamin C deficiency (*p* = 0.177).

The current evidence has shown that patients with vitamin C deficiency may experience more benefits from vitamin C supplementation than non-deficient patients (52). Yet, differences in study design may partially explain the inconsistencies of the effects on clinical outcomes. According to the present study results, the onset of vitamin C consumption may develop before ICU admission. These findings have important implications. Therefore, it is reasonable to propose future studies that evaluate the efficacy of vitamin C administration in earlier phases of COVID infection.

Interestingly, vitamin C levels increased from undetectable to low range values on day 5 of ICU admission in a subgroup of patients who had not received exogenous vitamin C supplementation during their ICU stay. These patients presented a single organ dysfunction, and shorter ICU stays. Consequently, a new hypothesis emerges to evaluate the potential benefits of vitamin C supplementation for reducing the severity of respiratory dysfunction and improving outcomes in future studies.

However, it is necessary to consider the results obtained so far in those recent studies evaluating vitamin C treatment, which have not been conclusive or have even yielded contrary conclusions. Rosengrave et al. (53) indicated that intravenous vitamin C did not provide significant decreases in the mean dose or duration of vasopressor infusion even though the population with severe organ dysfunction and high dependence on vasopressors has again deficient levels of vitamin C. The CITRIS-ALI trial (23) was a randomized, double-blind, placebo-controlled, multicenter trial conducted in 7 medical ICUs in the United States, enrolling patients (*N* = 167) with sepsis and ARDS present for less than 24 h; plasma ascorbate levels at enrollment were marginally deficient in both groups but a 96-h infusion of vitamin C compared with placebo did not significantly improve organ dysfunction scores or alter markers of inflammation and vascular injury. Hwang et al. (54) enrolled a total of 111 septic shock patients where serum levels and deficiency rates of vitamin C and thiamine during the first 72 h from enrolment are quite high in all study groups; but there was no significant difference in ΔSOFA scores between the treatment group and the placebo group. The VICTAS trial (55), among 501 participants randomized treatment with vitamin C, thiamine, and hydrocortisone, compared with placebo, did not significantly increase ventilator- and vasopressor-free days within 30 days. One of the last reports, the LOVIT trial (56), again showed vitamin C deficiency at the beginning of the study, however concluded that in adults with sepsis receiving vasopressor therapy in the ICU, those who received intravenous vitamin C had a higher risk of death or persistent organ dysfunction at 28 days than those who received placebo.

In the same way as our findings, it seems that in critically ill patients or those with a certain degree of organ dysfunction, the vitamin C deficiency occurs before the diagnosis of the clinical condition. This means that before diagnosing sepsis or ARDS, the plasma vitamin C levels are somehow already reduced.

Applying a precision medicine by identifying patients who could receive vitamin C by measuring plasma levels could be a priority. Some issues regarding the role of vitamin C in critically ill COVID-19 patients are pending to be addressed in clinical studies. Future studies should address whether all critically ill COVID-19 patients could benefit from vitamin C adjuvant treatment regardless of their vitamin C plasma concentrations or whether measuring plasmatic levels of vitamin C is beneficial before starting treatment.

In addition, Stoppe et al. (57) recently considered which are the future directions in the study of vitamin C. Among other aspects, they wonder if treatment with vitamin C should be used in patients with vitamin C deficiency, and therefore it seems relevant to establish when this deficit occurs.

This study has some limitations. First, this is a single-center study including a small sample of patients with no control group. Thus, the findings cannot be extrapolated to other ICU settings and should be confirmed in larger comparative studies. Second, the patient inclusion process was not consecutive. However, it unlikely changed the results.

## Conclusion

This observational study showed vitamin C plasma levels were undetectable on ICU admission in 86% of patients with acute respiratory failure due to COVID-19 pneumonia requiring respiratory support. This finding remained consistent throughout the study period.

## Data availability statement

The original contributions presented in the study are included in the article/supplementary material, further inquiries can be directed to the corresponding author.

## Ethics statement

The studies involving humans were approved by Clinical Research Ethics Committee (CEIm) of Vall d’Hebron University Hospital [PR(AG)687/2020]. The studies were conducted in accordance with the local legislation and institutional requirements. The ethics committee/institutional review board waived the requirement of written informed consent for participation from the participants or the participants' legal guardians/next of kin because the informed consent was waived due to epimediological situation, in between COVID-19 outbreak.

## Author contributions

LC-C: Conceptualization, Formal analysis, Investigation, Methodology, Project administration, Resources, Writing – original draft. JR-R: Conceptualization, Supervision, Writing – review & editing. EP-M: Writing – review & editing. LM: Writing – review & editing. IB: Writing – review & editing. CM-R: Writing – review & editing. CP: Writing – review & editing. RF-C: Writing – review & editing. SC: Writing – review & editing. YV-O: Writing – review & editing. VR: Writing – review & editing. AR-S: Writing – review & editing. MP: Writing – review & editing. RF: Writing – review & editing.

## Figures and Tables

**Table:** 

AKI	Acute Kidney Injury
APACHE	Acute Physiology and Chronic Health disease Classification System
ARDS	Acute Respiratory Distress Syndrome
CKD	chronic kidney disease
COPD	chronic obstructive pulmonary disease
COVID-19	Coronavirus infectious disease 2019
CRP	C-Reactive Protein
CRRT	Continuous renal replacement therapy
DD	D-Dimer
dL	deciliter
DM-II	type 2 diabetes mellitus
ECMO	extracorporeal membrane oxygenation
HFNC	high flow nasal cannula
ICU	Intensive Care Unit
IL	interleukin
IQR	Interquartile range
mg	milligrams
PaO_2_/FiO_2_ ratio	arterial oxygen pressure (P_a_O_2_), inspired fraction of oxygen (F_I_O_2_)
PF	PaO_2_/FiO_2_ ratio: arterial oxygen pressure (P_a_O_2_), inspired fraction of oxygen (F_I_O_2_)
ROS	reactive oxidative species
SARS-CoV-2	severe acute respiratory syndrome coronavirus 2
SOFA	Sequential Organ Failure Assessment
VAP	Ventilator-associated pneumonia
